# The Determinants of Length of Homeless Shelter Stays: Evidence-Based Regression Analyses

**DOI:** 10.3389/ijph.2021.1604273

**Published:** 2022-01-28

**Authors:** Haijing Hao, Monica Garfield, Sandeep Purao

**Affiliations:** ^1^ Department of Computer Information Systems, Bentley University, Waltham, MA, United States; ^2^ Department of Information and Process Management, Bentley University, Waltham, MA, United States

**Keywords:** disability, homeless shelter, length of stay, HMIS, regression

## Abstract

**Objective:** To identify determinants that contribute to the length of homeless shelter stay.

**Methods:** We utilized a unique dataset from the Homeless Management Information Systems from Boston, Massachusetts, United States, which contains 44,197 shelter stays for 17,070 adults between Jan. 2014 and May 2018.

**Results:** Our statistical analyses and regression model analyses show that factors that contribute to the length of a homeless shelter stay include being female, senior, disability, being Hispanic, or being Asian or Black African. A significant fraction of homeless shelter stays (76%) are experienced by individuals with at least one of three disabilities: physical disability, mental health issues, or substance use disorder. Recidivism also contributes to longer homeless shelter stays.

**Conclusion:** The results suggest possible program and policy implications. Several factors that contribute to longer homeless shelter stay, such as gender, age, disability, race, and ethnicity, may have funding implications. Age may point to the need for early interventions. Disability is developmental and may benefit from treatment and intervention. Finally, we find that length of stay and recidivism are not independent, and may form a vicious cycle that requires additional investigation.

## Introduction

Public health is concerned with the protection and improvement of the health of people and their communities. By some estimates, 80% of an individual’s health outcomes are due to social determinants [1], such as poverty, social exclusion, and poor health systems [2]. In recent years, poor housing and homelessness have emerged as another important public health concern [3, 4]. Homelessness is a widespread, complex social problem [5] that negatively impacts public health [6, 7] and healthcare systems [8, 9]. In 2020, the estimated number of homeless in the United States was 580,000. For the fourth consecutive year, homelessness increased nationwide [3]. Of those experiencing homelessness, 39.4% were African American, although they comprise just 13% of the US population [3], and 38.5% were female [3], but they experienced longer shelter stays [10]. With the impact of COVID-19 on the economy, and as the protection against housing evictions (section 361 of the Public Health Service Act) expires (June 2021), further increases are imminent [11]. The CARES Act of 2021 brought some relief for those who may be on the cusp of living with housing instability (the Emergency Rental Assistance program that makes $25 billion available to states, US territories, local governments, and Indian tribes) but it is set to expire December 2021 [12].

The increase in the number of people experiencing homelessness [3] is associated with significant healthcare costs. In one study it was reported that the hospitalization and emergency room expenditures by the homeless were 3.8 times the rate of an average Medicaid recipient [13]. Poor health has been identified as a contributor to homelessness, and vice versa [14]. Homeless persons have a lower quality of life compared to those who are not homeless [15, 16]. Contemporary scholarship has explored the inter-relationship between health and housing [17], and how healthcare services and funding agencies can find ways to better support the homeless population [13].

One early indicator of these healthcare costs is the cost of shelter services, $2,100 per person per month [18]. These shelters represent the first line of defense for the homeless. Emergency shelters (such as the ones included in this study) provide access to a variety of programs to help individuals reduce the length of time they experience homelessness. It has been established that those experiencing homelessness also experience disproportionately higher rates of premature mortality and poor physical and mental health status [19, 20]. A greater understanding of factors that contribute to the length of stay in a shelter can, therefore, have useful implications for policies and programs that impact public health. Some factors include which programs an individual is eligible for (for instance some programs are targeted for veterans, and others may be targeted for people with substance use disorder). While the length of a shelter stay provides some insight into the living experience of someone enduring homelessness it does not tell the full picture. If an individual no longer sleeps at a homeless shelter they may be sleeping on the streets and therefore be exposed to a number of potential issues including the impact of weather on their sleeping area or being vulnerable to other living beings who may wish to do them. In these cases a longer length of stay in a homeless shelter may be preferable to sleeping outside. However, if an individual is no longer sleeping in an emergency shelter due to their ability to find more stable sleeping and living arrangements the length of stay in a homeless shelter should be as short as feasible. In this paper, we analyze data from the HMIS data set and provide empirical evidence based on this data, which may be useful for prioritizing shelter services, and in turn, contribute to better public health outcomes.

## Methods

### Homeless Management Information Systems

The HEARTH Act, enacted on May 20, 2009, requires that U.S. Department of Housing and Urban Development (HUD) funded recipients and sub-recipients use Homeless Management Information Systems to collect data on individuals experiencing homelessness. A Homeless Management Information System (HMIS) is a local management information system used to collect client-level data and data on the provision of housing and services to homeless individuals and families who are at risk of homelessness. Each Continuum of Care (CoC) is responsible for selecting an HMIS software solution that complies with HUD’s data collection, management, and reporting standards. According to the authors’ knowledge, our study is the first—or one of the first—to examine the HMIS data at the individual level in Boston, MA, United States.

The data for the present study were acquired from Boston HMIS via our community partners and the City of Boston in August of 2020. It represents a compilation of records contributed from several agencies in the CoC and maintained within the HMIS. The period covered by the data is from January 1, 2014 to May 31, 2018. The data structure follows mandated reporting requirements and includes data such as Client, Enrollments, Exit, Services, Disability, Project, and other tables. The unique client Id, generated when an individual enrolls in a homeless service or project for the first time, anchors the information. Each time a client enrolls in a homeless service or project, an enrollment Id is created in the Enrollment table. When a client exits the service or project, an exit date is recorded for the enrollment Id in the Exit table. We use client Id, enrollment Id, and entry and exit date to compute the length of stay of each enrollment of each client, which we call a homeless shelter stay. For each completed homeless shelter stay within the dataset, the length of the stay was computed by subtracting the entry date from the exit date. We did not include stays that had no exit date or were ongoing on the last date of our dataset. A client may have multiple homeless shelter stays with the same or different services or projects (e.g., homeless shelters). Before we could move to analysis, the data was cleansed to remove missing or inconsistent information. The cleansed dataset included client and enrollment Id as well as demographics, disability status, project Ids, and entry and exit dates for 44,197 homeless shelter stays for 17,070 clients who were 16 years old or above.

### Statistical Methods

First, we conducted descriptive statistics of the dataset to have a big picture of the demographic distributions, and visualized the data by various charts. We presented the number of homeless shelter stays per individual experienced by histogram, exhibited the number of days per homeless shelter stay by histogram, and visualized the number of days of each homeless shelter stay by different age group.

To study which factors affect the length of homeless shelter stays, the average days of a homeless shelter stay by different factors were compared either by using a two-sample t-test with unequal variances for binary variables, such as female vs. male, disability vs. non-disability, or by using a one way ANOVA test for categorical risk factors such as race groups or age groups with equal variances, or using Kruskal–Wallis test if the ANOVA’s equal variance assumption is rejected by Bartlett’s test. All statistical analyses were applied to both the entire dataset for all age groups and the four sub-datasets for the four age groups. All analyses were performed using Stata SE 16.

To explore risk factors that impact the length of each homeless shelter stay an adult experienced, we constructed a set of log-linear regression models by taking logs of the dependent variable. Unlike conventional linear regression models, which examine factors that affect the dependent variable by the unit in the *length* of stay [21], our models examine factors that affect the dependent variable’s *relative change in the length* of stay. Also, taking logs of the dependent variable can adjust the abnormality among the dependent variable values, if there would be any. We also added interaction terms among independent variables to our models to account for the possibility that a factor may be heightened or dampened, when combined with other factors. We selected the final models based on the number of significant independent variables impacting our dependent variable by using a stepwise forward, and then stepwise backward selection method. The experimental regression analyses were performed with Stata 16.

## Results

### Descriptive Statistics

Among the 17,070 adult individuals, 74.2% were male and 25.8% were female. About 54% were White, 41.6% were Black African American, and the remaining were other race groups, e.g., American Indian, Native Hawaii Islanders, Asian, and multi-race, which accounted for less than 2% each. About 18% of the individuals reported they were Hispanic. We categorized the individuals into four age groups: between 16 and 24 years old, between 25 and 49 years old, between 50 and 64 years old, and 65 years old or above. Individuals in the 25 to 49 age group were the largest group (61%), the second-largest (25%) was individuals between 50 and 64, followed by younger adults (10.4%), i.e., individuals between 16 and 24, and finally, seniors (3.9%), i.e., individuals 65 years old and above. Both the median and the average age on the first entry into a shelter were about 41 years, and the maximum age was 93 years.

**FIGURE 1:**
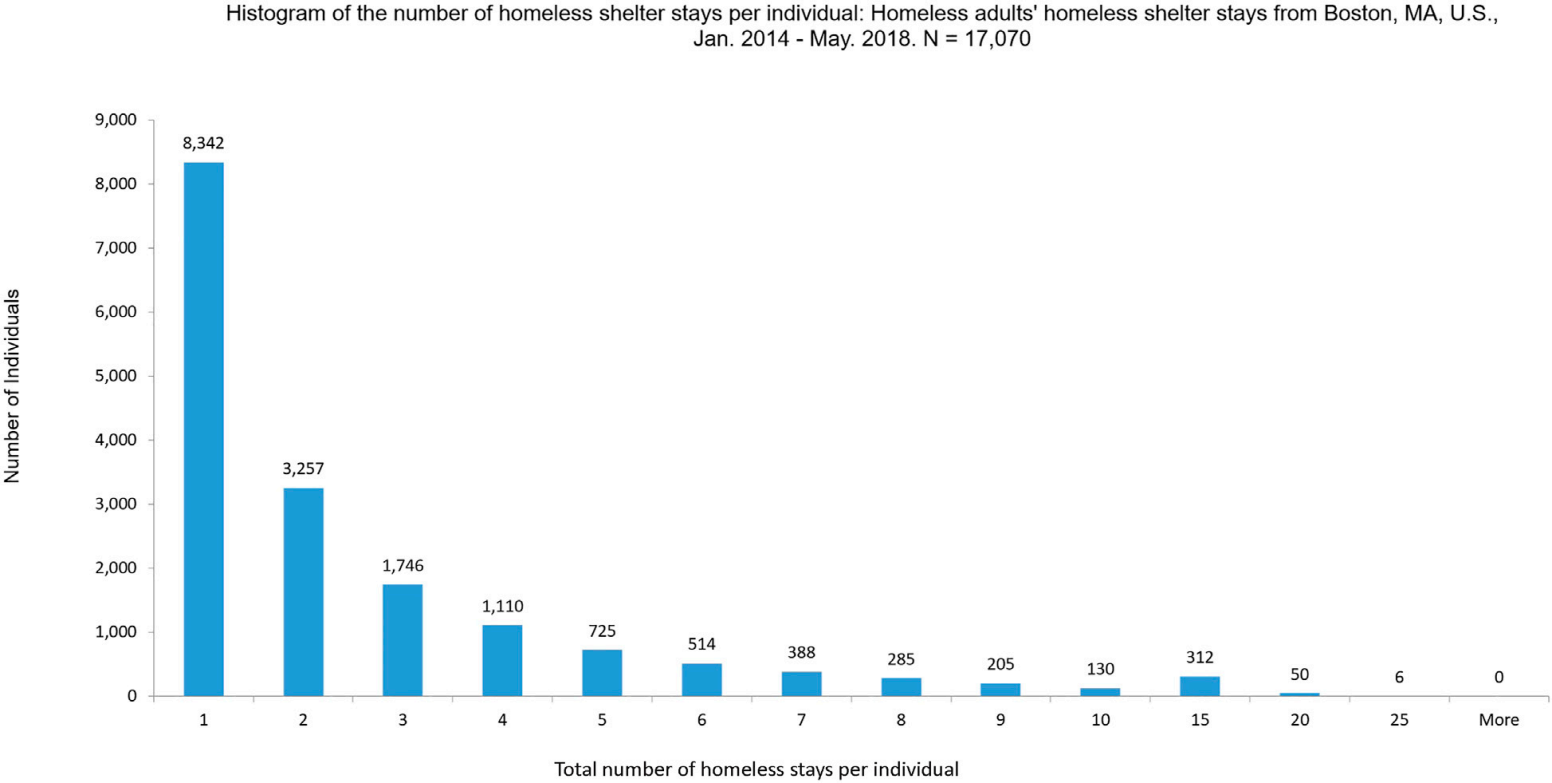
Histogram of the total number of homeless shelter stays per individual: Homeless adults’ homeless shelter stays from Boston, MA, United States, January 1, 2014–May 31, 2018. *n* = 17,070.

On average, an individual from this data set experienced ∼2.6 homeless shelter stays during the time period we examined, with a median of 2 and a maximum of 24. We define recidivism status as a client with more than one homeless shelter stay. We code this client’s recidivism status as 1, otherwise, the recidivism status is coded as 0 which means this client only has one homeless shelter stay. Figure 1 shows the histogram of the number of homeless shelter stays experienced by all individuals. From Figure 1, we can see of the 17,070 individuals, 8,342 (∼49%) experienced one homeless shelter stay, whereas 8,728 (∼51%) experienced two or more homeless shelter stays. Among those who experienced two or more homeless shelter stays, 3,257 (∼37%) faced two homeless shelter stays, 2,615 (∼30%) individuals experienced five or more homeless shelter stays, and 498 (∼5.7%) experienced 10 or more homeless shelter stays in these 53 months, pointing to high recidivism.

**FIGURE 2:**
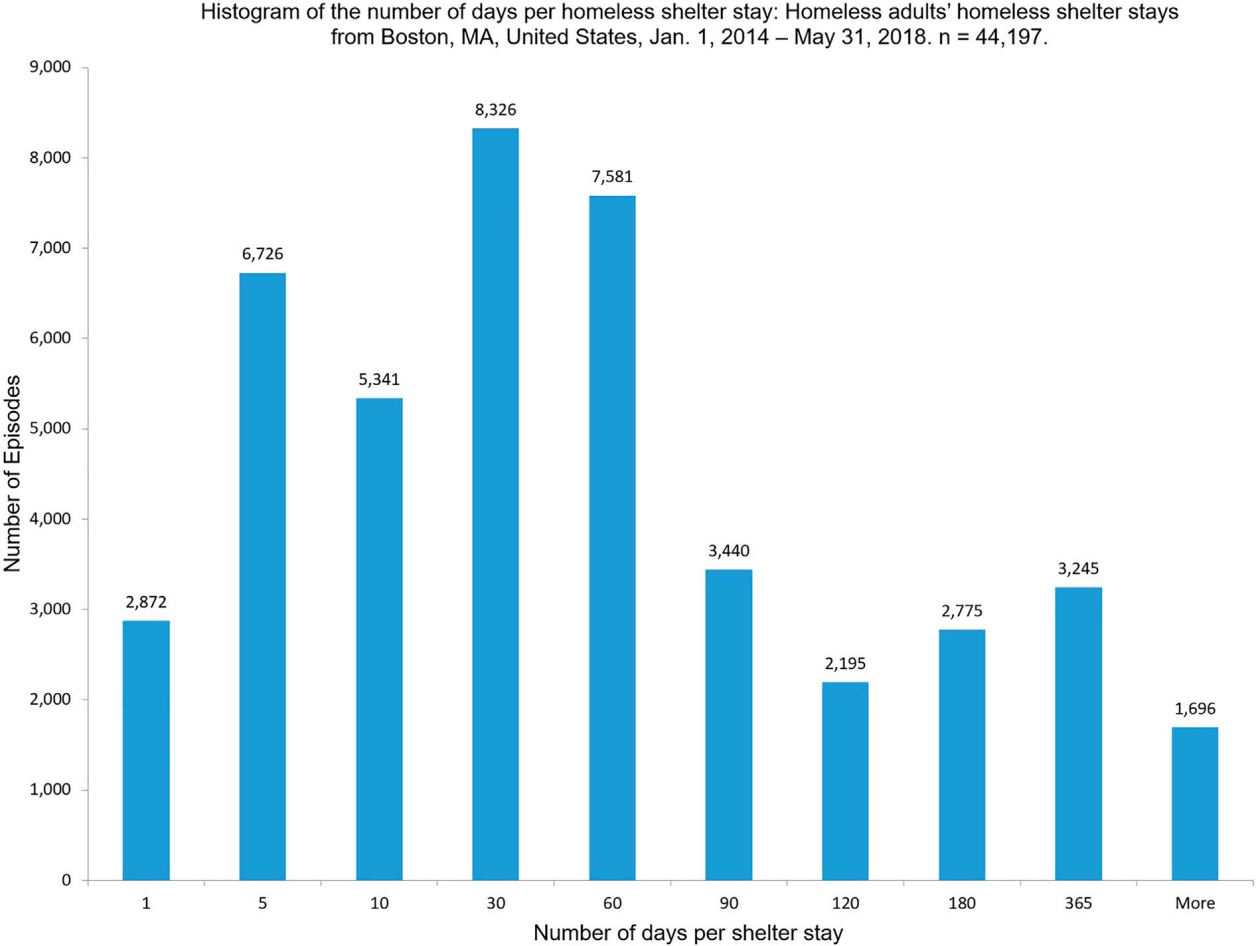
Histogram of the number of days per homeless shelter stays: Homeless adults’ homeless shelter stays from Boston, MA, United States, January 1, 2014–May 31, 2018. *n* = 44,197.

The length of each homeless shelter stay also varied significantly across individuals over time. Among the 44,197 homeless shelter stays, on average, a homeless shelter stay was about 77 days, with the median 30 days, and the maximum of 5,030 days (the entry date started in 2002 for this extreme case). As Figure 2 shows, 2,872 (∼6.5%) homeless shelter stays were just 1 day long, 6,726 homeless shelter stays (∼15%) were between 2 days and 5 days, and 34,695 homeless shelter stays (78.5%) were 10 days or longer. About 81% of all homeless shelter stays were by clients who have experienced recidivism.

**FIGURE 3:**
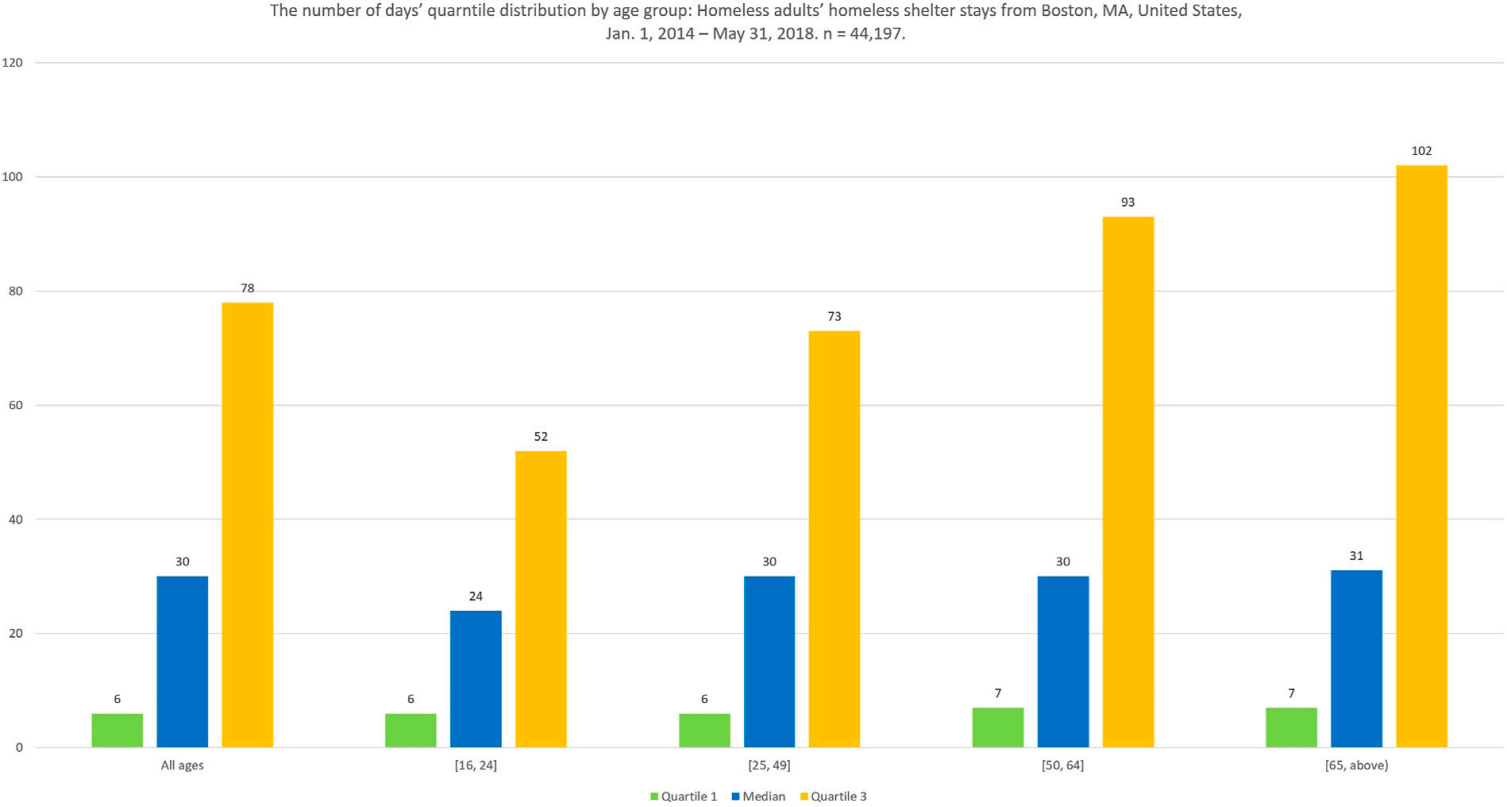
The number of days’ quartile distribution by age group: Homeless adults’ homeless shelter stays from Boston, MA, United States, January 1, 2014–May 31, 2018. *n* = 44,197.

The number of days of each homeless shelter stay also varied greatly by different age groups, as Figure 3 exhibits the first quartile, the median, and the third quartile number of days of different age groups. We can see the median number of days of different age groups increases as the age increases.

Disability status was not reported as a part of demographics but captured at each enrollment into a shelter service because it may change over time. Disability status indicates that an individual has at least one of three disabilities: physical disability, mental health issues, or substance use disorder. Of the 44,197 homeless shelter stays, 33,590 (∼76%) were experienced by individuals who were recorded to have at least one of the three disabilities at one enrollment. We also calculated whether an individual had ever reported having had at least one of the three disabilities in any homeless shelter stay. If they did, the individual’s disability status was set to 1. Using this calculation, 13,282 of the 17,070 (∼78%) individuals in this dataset reported at least one disability at a minimum of one point in time.

### Statistical Analyses

Table 1 presents the descriptive statistics and statistical analysis results. All Bartlett’s tests for categorical variables were statistically significant. Hence we used Kruskal–Wallis test for categorical variables, such as race group and age group, and the *p*-values for the categorical variable are the *p*-values from Krushak-Wallis tests. The *p*-values for the binary variables are from two-sample t-tests. We can see that the average days per homeless shelter stay between disability and no-disability group have a statistically significant difference in most age groups (the difference is 9.03 with *p* < 0.0001 for all age groups, the difference is 11.32 with *p* = 0.0014 for age 50 and 64 years old, and the difference is 39.61 with *p* < 0.0001 for 65 years old or above). The average days per homeless shelter stay between one-time visitors and multiple time visitors (recidivism) are also statistically significant for three age groups, age between 25 and 49 years old, age between 50 and 64 years old, and age 65 years old or above. But interestingly, we can see that the adults in the 25 and 49 years old group, have the average length per homeless shelter stay for multiple time visitors of about 7 days longer than for one time visitors (*p* = 0.0028), but the other two age groups are the opposite in that the multiple time visitors’ average stay are shorter than one time visitors (*p* < 0.0001 for age between 50 and 64 years old, and *p* = 0.014 for 65 years old or above). The Kruskal-Wallis test for categorical race variable shows statistical significance for the overall population (*p* < 0.0001), age between 25 and 49 years old (*p* = 0.0001), and age between 50 and 64 (*p* = 0.0001). The Kruskal-Wallis test for categorical age group variable also shows statistical significance (*p* = 0.0001) which means average days per homeless shelter stay in each age group’s distribution is not equal.

**TABLE 1:** The average days of adults’ homeless shelter stay by risk factors in Boston, Massachusetts, United States, January 1, 2014–May 31, 2018.

Risk factors	All ages (n = 44,197)			Age 16–24 years old (n = 2,645)			Age 25–49 years old (n = 25,272)			Age 50–64 years old (n = 14,287)			Age 65 years old or above (n = 1,993)		
	n (%)	Mean	Difference between two means	n (%)	Mean	Difference between two means	n (%)	Mean	Difference between two means	n (%)	Mean	Difference between two means	n (%)	Mean	Difference between two means
			(*p*-Value)			(*p*-Value)			(*p*-Value)			(*p*-Value)			(*p*-Value)
Gender															
Female	9,757 (22.08)	77.69	1.30 (0.462)	921 (34.82)	56.74	3.34 (0.430)	5,491 (21.73)	69.97	0.68 (0.745)	2,872 (20.10)	92.94	3.62 (0.310)	473 (23.73)	115.45	7.00 (0.180)
Male	34,440 (77.92)	76.39		1,724 (65.18)	53.40		19,781 (78.27)	69.29		11,415 (79.90)	89.32		1,520 (76.27)	97.75	
Veteran status															0.512
Veteran	3,043 6.89)	71.56	−6.50 (0.075)	36 (1.36)	60.94	6.47 (0.679)	982 (3.89)	55.58	−14.42 (<0.0001*)	1,693 (11.85)	77.03	−14.76 (0.0002*)	332 (16.66)	92.09	−11.83 (0.512)
Non-Veteran	41,154 (93.11)	77.06		2,609 (98.64)	54.47		24,290 (96.11)	70.00		12,594 (88.15)	91.79		1,661 (83.34)	103.92	
Ethnicity															
Hispanic	8,023 (18.15)	77.57	1.09 (0.563)	574 (21.70)	55.35	1.01 (0.846)	5,142 (20.35)	71.02	1.98 (0.346)	2,065 (14.45)	95.57	6.46 (0.159)	242 (12.14)	116.01	6.01 (0.201)
Non-Hispanic	36,174 (81.85)	76.48		2,071 (78.3)	54.34		20,130 (79.65)	69.04		12,222 (85.55)	89.11		1,751 (87.86)	100.00	
Disability status															
Disability	33,467 (75.72)	78.87	9.03 (<0.0001*)	1,613 (60.98)	53.31	−3.22 (0.449)	18,752 (74.20)	70.21	2.98 (0.112)	11,576 (81.02)	92.19	11.32 (0.0014*)	1,526 (76.57)	111.23	39.61 (<0.0001*)
No-Disability	10,730 (24.28)	69.84		1,032 (39.02)	56.53		6,520 25.80)	67.23		2,711 (18.98)	80.87		467 (23.43)	71.62	
Recidivism Status															
Enrolled Multiple Times	35,855 (81.13)	75.98	−3.69 (0.122)	1,797 (67.94)	55.39	2.59 (0.539)	20,596 (81.50)	70.82	7.43 (0.0028*)	11,934 (83.53)	85.93	−24.96 (<0.0001*)	1,528 (76.67)	92.07	−42.35 (0.014*)
Enrolled One Time	8,342 (18.87)	79.67		848 (32.06)	52.80		4,676 (18.50)	63.39		2,353 (16.47)	110.89		465 (23.33)	134.42	
Races															
American Indian Native	377 (0.85)	76.11	<0.0001*	14 (0.53)	65.07	0.6786	206 (0.82)	68.36	0.0001*	140 (0.98)	81.86	0.0001*	17 (0.85)	131.65	0.2920
Asian	594 (1.34)	89.03		52 (1.97)	60.5		352 (1.39)	83.32		147 (1.03)	121.16		43 (2.16)	60.42	
Black African American	19,120 (43.26)	82.31		1,293 (48.89)	54.86		10,635 (42.08)	76.35		6,381 (44.66)	95.96		811 (40.69)	96.91	
Native Hawaii Pacific	453 (1.03)	73.29		33 (1.25)	34		275 (1.09)	71.01		136 (0.95)	86.43		9 (0.45)	88.33	
Multi race	400 (0.91)	73.03		48 (1.81)	62.23		234 (0.93)	64.53		104 (0.73)	99.46		14 (0.70)	55.93	
White	23,253 (52.61)	71.87		1,205 (45.56)	54.12		13,570 (53.70)	63.74		7,379 (51.65)	84.40		1,099 (55.14)	107.53	
Age group (years)															
Age_16_24	2,645 (5.98)	54.56	0.0001*												
Age_25_49	25,272 (57.18)	69.44													
Age_50_64	14,287 (32.33)	90.04													
Age 65 or above	1,993 (4.51)	101.95													

Note: * indicates the statistical significance at 5% level.

Note: If a two-sample t-test with unequal variance is performed for a binary variable, the column for *p*-value will show both the difference between means and the *p*-value. If a Kruskal-Wallis test is performed for a categorical variable, the column for *p*-value will only show the *p*-value from the Kruskal-Wallis test.

### Regression Analyses

Table 2 shows four regression model results, and Model 4 represents the best model for understanding which factors and their interactions affect the length of homeless shelter stay, based on the adjusted R-squared. AIC and BIC also present similar model fit results.

**TABLE 2:** Regression analysis of adults’ homeless shelter stays in Boston, MA, United States, January 1, 2014 to May 31, 2018 (*n* = 44,197).

Factors	Model 1	Model 2	Model 3	Model 4
	Coef. (95% CI)	Coef. (95% CI)	Coef. (95% CI)	Coef. (95% CI)
Female	−0.01 (−0.05, 0.03)	0.003 (−0.03, 0.04)	0.20*(0.15, 0.25)	0.37* (0.20, 0.54)
Veteran	−0.15*(−0.22, −0.09)	−0.14*(−0.20, −0.08)	−0.10*(−0.17, −0.04)	−0.10* (−0.16, −0.04)
Hispanic	0.09*(0.05, 0.13)	0.09*(0.05, 0.14)	0.10*(0.06, 0.14)	0.09* (0.06, 0.13)
Race				
American Indian Native	0.15 (−0.02, 0.32)	0.15 (−0.02, 0.32)	0.16 (−0.004, 0.32)	0.15 (−0.01, 0.31)
Asian	0.21*(0.08, 0.35)	0.21*(0.08, 0.35)	0.27*(0.14, 0.40)	0.27*<(0.14, 0.40)
Black African American	0.16*(0.12, 0.19)	0.15*(0.12, 0.18)	0.15*(0.12, 0.18)	0.15*(0.12, 0.18)
Native Hawaii Pacific	−0.004 0.16, 0.15)	−0.02 (−0.16, 0.16)	−0.02 (−0.17, 0.13)	−0.02 (−0.17, 0.13)
Multi race	0.1 (−0.06, 0.26)	0.10 (−0.07, 0.26)	0.10 (−0.06, 0.26)	0.10 (−0.06, 0.25)
Age				
Age_16_24	−0.43*(−0.53, −0.34)	−0.52*(−0.51, 0.32)	−0.51*(−0.60, −0.42)	−0.28*(−0.46, −0.09)
Age_25_49	−0.22*(−0.30, −0.14)	−0.23*(−0.30, −0.15)	−0.27*(−0.35, −0.20)	−0.02 (−0.18, 0.14)
Age_50_64	−0.08*(−0.16, −0.002)	−0.09*(−0.17, −0.01)	−0.12*(−0.19, −0.04)	0.06 (−0.11, 0.22)
Disability	0.01 (−0.02, 0.05)	−0.003*(−0.04, 0.03)	0.09*(0.05, 0.12)	0.32*(0.16, 0.49)
Recidivism Status	-	−0.22*(0.18, 0.26)	−0.21*(0.17, 0.25)	−0.21*(0.17, 0.25)
Project fixed effects	No	No	Yes	Yes
Gender and age interaction				
Female * Age_16_24	-	-	-	−0.23*(−0.44, −0.02)
Female * Age_25_49	-	-	-	−0.20*(−0.37, −0.02)
Female * Age_50_64	-	-	-	−0.13 (−0.30, 0.05)
Disability and age interaction				
Disability * Age_16_24	-	-	-	−0.23*(−0.43, −0.02)
Disability * Age_25_49	-	-	-	−0.27*(−0.44, −0.10)
Disability * Age_50_64	-	-	-	−0.19*(−0.37, −0.01)
Constant	3.24*(3.16, 3.32)	3.08*(3.00, 3.17)	1.90*(1.68, 2.13)	1.70*(1.43, 1.96)
Adjusted R-squared	0.0061	0.0087	0.0841	0.0844
AIC	169,852.2	169,738.5	166,257.6	166,251.7
BIC	169,965.2	169,860.3	166,535.8	166,582.1

Note: * indicates the statistical significance at 5% level.

Note: Male, non-veteran, non-hispanic, White, Age 65 and above, no-disability, and one-time visitor are the reference groups for binary or categorical variables in Table 2 accordingly.

From Table 2, we see female, veteran status, Hispanic, Asian, Black, age groups, and disability status are statistically significant in most models. Model 4 shows that not only female (b = 0.37, t = 4.27, *p* < 0.0001), veteran status (b = -0.10, t = −3.05, *p* = 0.002), Hispanic (b = 0.09, t = 4.71, *p* < 0.0001), Asian (b = 0.27, t = 4.06, *p* < 0.0001), Black African American (b = 0.15, t = 9.57, *p* < 0.0001), young adults whose age are between 16 and 24 years old (b = −0.28, t = −2.86, *p* = 0.004), disability status (b = 0.32, t = 3.80, *p* < 0.0001), and recidivism status (b = 0.21, t = 10.58, *p* < 0.0001) have statistical significant impacts on the log of the length of homeless shelter stay, but also the interaction terms, between gender and 16–24 years old (b = −0.22, t = −2.12, *p* = 0.034), between gender and 25 and 49 years old (b = −0.20, t = −2.23, *p* = 0.026), between disability and 16–24 years old (b = −0.23, t = −2.13, *p* = 0.033), between disability and 25–49 years old (b = -0.27, t = −3.06, *p* = 0.002), and between disability and 50–64 years old (b = −0.19, t = −2.12, *p* = 0.034) have statistical significant impacts on the log of the length of the homeless shelter stay. These interaction effects point out that the effects of gender and disability are differentiated across age groups. A positive coefficient, being calculated by including both the independent variable coefficient and the interaction term coefficient, indicates that the factor is associated with a relatively longer homeless shelter stay than its reference group. So, being female, having at least one disability, being Hispanic, being Asian, being Black African American, and experiencing recidivism in homelessness have relatively longer homeless shelter stays than their reference groups, being male, having no disability, being non-Hispanic, being White, and being a one-time visitor. A negative coefficient indicates that the risk factor is associated with a relatively shorter homeless shelter stay than its reference group, such as being veteran and being younger (age between 16 and 24 years old) than their reference groups, being non-Veteran and being 65 years old or above, respectively.

In addition, both Model 3 and Model 4 also controlled for the homeless projects, i.e., different shelters, potentially with access to different services, as project fixed effects. Almost all projects were statistically significant in both models. An investigation of the relative effectiveness of each homeless shelter and its impact on the length of homeless shelter stay will need a separate study with more information.

## Discussion

This study reports a series of statistical analyses and regression model analyses based on the HMIS data from Boston, Massachusetts, the United States, from January 2014 to May 2018 to examine the factors that impact the length of homeless shelter stays in Boston, a large metropolitan area in the U.S.

We found that many demographic factors and their interactions have statistically significant impacts on the length of homeless shelter stays. Overall, women, seniors, Hispanics, Asians, Black African Americans, people with a disability (including a physical disability, mental health issue, or substance use disorder), and visitors that are experiencing recidivism have longer homeless shelter stays, compared to their counterparts: men, younger people, non-Hispanic, White, people without any disabilities, and one time visitors. Many additional factors may impact the length of a homeless shelter stay that our data does not cover due to data limitations, and the reasons for the impact of these factors are not clearly understood.

Although the effect varies over different age groups, in most age groups the length of homeless shelter stays for women remains relatively longer than the base category, men 65 years old or above group, from 15% (women in 25–49 years old group) to 37% (women above 65 years old group) longer. This finding aligns with prior studies that have shown that women stay in homeless programs significantly (74%) longer than men [10]. This is concerning as other work has reported that the rise in the number of unsheltered homeless women (12%) is outpacing that of unsheltered homeless men (7%) [3]. These findings also have cost implications. The per-person cost for first-time homeless women is about 97% higher than for men because of a higher need to provide privacy [10]. Addressing women’s homelessness status by decreasing the length of their homeless shelter stays can, therefore, also reduce the overall cost of homeless shelters, and contribute to improving important social determinants of health. However, we need to be aware of the vulnerability of women who experience homelessness and ensure that by lowering their length of stay in a shelter they are not put at increased risk or exposed to elements that may negatively impact their quality of life.

The U.S. is also an aging society. About 15% of the U.S. population is above 65 years old [22], and this number will continue to increase as people are living longer due to advances in health care. It is anticipated that the elderly homeless population will also grow [23]. Although in our data only about 3.9% of homeless individuals and 4.5% of the homeless shelter stays are elderly people who are 65 years or older, the results show that the elderly face significantly longer homeless shelter stays than all other age groups. As this population segment grows, so too will the costs associated with sheltering those experiencing homeless, and further exacerbate the demands on public health infrastructure.

A different pattern is seen for factors associated with race. Although Black African Americans represent only 13% of the U.S. population [3], many studies show that African Americans remain a disproportionately high fraction of those experiencing homelessness [3]. Among families experiencing homelessness in New York City, for example, about 56% were African American [24]. Our study shows that among all the adults experiencing homelessness in Boston, Massachusetts, 42% are Black African Americans who represent 25.2% of the population in the city [25], and about 43% of the homeless shelter stays in Boston were experienced by Black African American. Also, a Black African American faces homeless shelter stays that are, on average, 15% longer than those faced by a White person. This consistently higher percentage of homelessness and longer homeless shelter stays among Black African Americans, noted in many studies [26], points to continuing, pervasive racial inequality in those experiencing homelessness in the U.S. Also, our finding that Asians experience about 27% longer homeless shelter stay than Whites has not been reported in other studies. This finding echoes the persistent view that minority groups facing hardship remain vulnerable and face additional obstacles [27].

Disability status, including physical disability, mental health problems, and substance abuse, is another factor that is consistently statistically significant across our models. Our data shows that 79% of the individuals experiencing homelessness reported at least one of the three disabilities; and 76% of the homeless shelter stays were experienced by individuals who reported at least one of the three disabilities. The CoC Homeless Populations and Subpopulations Reports show that in Massachusetts, 33.6% of the homeless population suffered from severe mental illness or chronic substance abuse, while the national estimate is 37.8% [3]. Drug overdose is the leading cause of death nationwide among sheltered homeless people and the second-highest leading cause of death among all people experiencing homelessness, sheltered and unsheltered [28]. Mental health issues and substance use disorder have been salient problems for individuals experiencing homelessness for the first time as well as individuals who have experienced long-term homelessness, a pattern noted in the U.S. and elsewhere [9]. Many studies have discussed the relationship between substance abuse and homelessness. Sometimes substance abuse precedes homelessness but homelessness can also exacerbate the issue [4, 29, 30]. Higher healthcare costs among the homeless are mostly associated with diagnosed mental health disorders or substance abuse [31]. But disability status is different from other demographic variables; it is a developmental factor that can change from one homeless shelter stay to the next, and therefore, a potentially improvable factor. However, we need to be cautious on how these disabilities are treated or supported to ensure long-term improvement in quality of life and not just how they impact the length of stay in a homeless shelter by those with disabilities. Often times the treatment and support programs that target individuals with disabilities require long term effort and therefore may be the cause of a longer length in homeless stay in order to improve long term quality of life.

Recidivism is another factor that should receive attention because, on average, an individual that has visited a shelter more than once (in the time frame of our data set) would experience about a 21% longer homeless shelter stay than a first-time visitor. About 51% of all homeless individuals are ones experiencing recidivism, and about 81% of the homeless shelter stay in our data were experienced by those who were experiencing recidivism.

### Public Health Implications

Our findings point to several factors associated with the length of stay that deserves particular attention when considering policy implications. Direct efforts to address the disadvantaged subgroups, such as women, seniors, Asians, Black African Americans, and those that have experienced more than one homeless shelter stay when allocating funding, should be considered by shelter administrators and policymakers. Further, due to the impact of age on the length of the homeless shelter stay programs targeted to intervene early to prevent such age-associated homelessness need to be prioritized. Finally, policymakers need to address the needs of at-risk individuals by providing low-cost long-term support in areas of healthcare (including mental health and substance abuse). These intervention programs could include programs that treat disabilities, such as healthcare programs to improve physical disability, counseling sessions to improve mental health issues, and substance prevention or rehabilitation programs to treat substance use disorder. Such changes have the potential to decrease recidivism and in turn the long-term costs of caring for individuals who experience homelessness and improve the quality of life for these individuals.

### Limitations

This study has limitations. First, the source for our data is a compilation of records contributed from several agencies in the continuum of care in Boston, MA, and maintained within the HMIS in response to the federal government’s reporting requirements. Although this allows for a larger dataset (e.g., beyond a single homeless shelter), it contains some inherent weaknesses such as missing information or input errors. Second, the models we develop rely on variables from this dataset. The adjusted R-squared for our best model was only 0.0844. This is rather low, however, homelessness is a complex societal problem with significant data availability concerns. Progress can be made in small increments such as the one we have shown, with a model that includes parameters for which data is available, and are statistically significant. Further model development and refinement may benefit from additional variables drawn from other sources, such as individuals’ level of education, safety net indicators, community-level unemployment rates, or housing market data. Third, the observed association between the factors and the dependent variable (the log of the length of homeless shelter stay) in our models should be interpreted cautiously with full consideration of our dataset and the data context. Finally, this data only provides information about when an individual enters and exits a homeless shelter, not about where they go after they leave. It is possible that in some cases longer lengths of stay in a homeless shelter are preferable to the alternative that may include sleeping on the streets or in other unsafe locations. The models have the potential to be included within a triage effort that would allow individual homeless shelters to make more informed decisions about arriving guests based on the risk factors they report [32].

### Conclusion

Based on a dataset from Boston’s HMIS, including 17,070 individuals who experienced 44,197 homeless shelter stays, we develop a series of statistical analyses and a set of log-linear regression models with factors and their interactions to identify factors that may impact the length of homeless shelter stays. The model results show that women, seniors, people with disabilities, individuals experiencing recidivism, and individuals who are Asian or Black African American have longer homeless shelter stay on average than their counterparts in shelter services. Targeted programs and funding allocations that help these vulnerable sub-groups may be one approach to addressing the problem. A promising area to focus on is to increase direct efforts to address the individuals’ disabilities which are developmental factors and have the potential to reduce the length of homeless shelter stay. Addressing these risk factors can help improve the health of those homeless individuals and their communities as well as reduce the cost burden on healthcare systems. However, we need to recognize that an increase in length of stay for some populations experiencing homelessness (i.e., women, those with disabilities) may lead to a higher quality of life when the alternative is to sleep in a more risky location (on the streets, in a home with an unsafe household member).
